# Insights into plant biodiversity conservation in large river valleys in China: A spatial analysis of species and phylogenetic diversity

**DOI:** 10.1002/ece3.8940

**Published:** 2022-05-19

**Authors:** Xudong Yang, Fei Qin, Tiantian Xue, Changying Xia, Sudhindra R. Gadagkar, Shengxiang Yu

**Affiliations:** ^1^ State Key Laboratory of Systematic and Evolutionary Botany Institute of Botany Chinese Academy of Sciences Beijing China; ^2^ University of Chinese Academy of Sciences Beijing China; ^3^ 26463 School of Life Sciences Chongqing Key Laboratory of Plant Resource Conservation and Germplasm Innovation Southwest University Chongqing China; ^4^ 3541 Biomedical Sciences Program College of Graduate Studies Midwestern University Glendale Arizona USA; ^5^ 3541 College of Veterinary Medicine Midwestern University Glendale Arizona USA

**Keywords:** conservation gap, distribution pattern, diversity hotspot, plant diversity, river valley, spatial phylogenetics, species richness

## Abstract

Large river valleys (LRVs) are heterogeneous in habitat and rich in biodiversity, but they are largely overlooked in policies that prioritize conservation. Here, we aimed to identify plant diversity hotspots along LRVs based on species richness and spatial phylogenetics, evaluate current conservation effectiveness, determine gaps in the conservation networks, and offer suggestions for prioritizing conservation. We divided the study region into 50 km × 50 km grid cells and determined the distribution patterns of seed plants by studying 124,927 occurrence points belonging to 14,481 species, using different algorithms. We generated phylogenies for the plants using the “V. PhyloMaker” R package, determined spatial phylogenetics, and conducted correlation analyses between different distribution patterns and spatial phylogenetics. We evaluated the effectiveness of current conservation practices and discovered gaps of hotspots within the conservation networks. In the process, we identified 36 grid cells as hotspots (covering 10% of the total area) that contained 83.4% of the species. Fifty‐eight percent of the hotspot area falls under the protection of national nature reserves (NNRs) and 83% falls under national and provincial nature reserves (NRs), with 42% of the area identified as conservation gaps of NNRs and 17% of the area as gaps of NRs. The hotspots contained high proportions of endemic and threatened species, as did conservation gaps. Therefore, it is necessary to optimize the layout of current conservation networks, establish micro‐nature reserves, conduct targeted conservation priority planning focused on specific plant groups, and promote conservation awareness. Our results show that the conservation of three hotspots in Southwest China, in particular, is likely to positively affect the protection of biodiversity in the LRVs, especially with the participation of the neighboring countries, India, Myanmar, and Laos.

## Graphical abstract

We detected 10 diversity hotspots for conservation priority along six large river valleys (LRVs) in China by using numerous occurrence data, high‐resolution grid cell, and comprehensive conservation indicators. It is necessary to optimize the layout of current conservation networks, establish micro‐nature reserves, conduct targeted conservation priority planning focusing on specific plant groups, and promote conservation awareness. The conservation of three hotspots in Southwest China will be more effective in protecting biodiversity in the river valleys with the participation of neighboring countries India, Myanmar, and Laos.

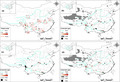

## 1 INTRODUCTION

The sustained good health and well‐being of life on earth is contingent upon a robust and biologically diverse environment. However, biodiversity in many regions is in sharp decline, largely due to human activity (Brooks et al., 2006; Di Marco & Santini, 2015; Newbold et al., 2015). Identification of “hotspots” is a popular approach in biodiversity conservation studies (Brooks et al., 2006; Brummitt & Lughadha, 2003; Myers et al., 2000). This approach appears to have gained traction when comprehensive methods and integrated indicators such as geographical distribution of species, taxonomic endemism, weighted endemism (WE) measures, and evolutionary information began to be employed in characterizing the biodiversity of a region (Cadotte & Davies, 2010; Huang et al., 2016; Redding & Mooers, 2006; Rosauer et al., 2009).

River valleys (Figure S1) traverse many ecological zones characterized by a change in altitude, abundant water and nutrient sources, heterogeneous habitats, diverse vegetation, and multiple ecosystems conducive to speciation and diversification, in addition to multiple endangered and relict species (Favre et al., 2015; Han et al., 2016; Zeng et al., 2018). Furthermore, they play a vital role by providing important pathways for migrating fish and dispersal of plants into new habitats (Andersson et al., 2000; Johansson & Nilsson, 1993; Merritt & Wohl, 2006). However, large‐scale anthropogenic perturbations, including industrial pollution, agricultural runoff, and sewage discharge, have intensified the eutrophication of river waters severely, killing native plants like hydrophytes and promoting rampant invasion by alien plants (Anderson & Garrison, 1997; Meybeck, 2003; Nixon, 1995; Zhang et al., 1999). Unfortunately, the crucial role of river valleys in generating biodiversity and preserving it appears to have largely been overlooked (Fonseca & Venticinque, 2018; Oliver et al., 2017). There is an urgent need for prioritizing large river valleys (LRVs) for their value in sustaining and conserving biodiversity and sustaining the livelihoods of indigenous peoples subsisting on the resources in the river valleys (Carrizo et al., 2017; Darwall et al., 2011).

However, there have been few studies aimed at understanding the distribution of plant species in river valleys, severely hindering the identification of priority areas for conservation. Most studies on river systems have focused on the rivers themselves, with emphasis on the temporal and spatial variations in water quality, eutrophication, and potability of water (Bu et al., 2019; Eliku & Leta, 2018; Vörösmarty et al., 2010). For example, some studies have focused on the effects on aquatic fauna, such as fish, reptiles, and amphibians (Mehta & Kushwaha, 2016; Wang et al., 2007), while others have considered the impacts on plant communities and ecosystems (Andersson et al., 2000; Carrizo et al., 2017; Merritt & Wohl, 2006). Thus, the role of river valleys in prioritizing conservation biodiversity hotspots is poorly understood and largely unknown.

China is home to several large rivers, such as Lancang (Mekong), Nujiang (Salween), Yarlung Zangbo (Brahmaputra), Changjiang (Yangtze), and Huanghe (Yellow), that rise in the Himalayas and circumjacent areas. These rivers then flow out from the vast Qinghai‐Tibet plateau, and along areas known for high biodiversity, and form important drainage systems in East Asia (Zhang et al., 2011). The drainage basins of these rivers display enormous differences in geographical, climatological, and topographical features and are home to thousands of important plant groups (López‐pujol et al., 2006). Despite this, however, river valleys tend to be neglected in studies of biodiversity conservation. Exacerbating the situation, China has a very large number of threatened species, with 3879 species on the list of threatened plant species, accounting for 10.84% of the native higher plant species, largely due to overexploitation, habitat loss, and fragmentation (Qin et al., 2017).

Studies have shown that incorporating large and comprehensive datasets along with multiple indicators, such as species richness, taxonomic endemism, and phylogenetic diversity (PD) (Huang et al., 2012, 2016; Luo et al., 2016; Yang et al., 2021), may provide insights for conservation priority planning. In this study, we used about 125,000 occurrence points and multiple conservation indicators to detect the distribution patterns of diversity hotspots along six large river valleys in China. Specifically, we aimed to (1) reveal the distribution patterns of species richness, endemism, conservation status, and PD of plants in the LRVs and test the congruence between each pair of indices; (2) assess the conservation effectiveness of current conservation networks, focusing on hotspots and identify conservation gaps; and (3) present recommendations and suggestions for effective strategies for conservation.

## 2 METHODS

### 2.1 Representative LRVs

According to “Measures For Classification of River Courses” (issued by Ministry of Water Resources of the People's Republic of China in 1994) and Liu (2014), large rivers are characterized by superlatives in terms of length, drop, basin area, and volume of runoff, all of which play an important role in preserving biodiversity, especially in light of the ongoing relentless, albeit necessary, anthropogenic change. On this basis, six large rivers in China were selected for the study, namely Changjiang, Huanghe, Lancang, Nujiang, Yarlung Zangbo, and Zhujiang (Pearl). These rivers are confined to the south and southwest of China, known for the aggregation of high numbers of endemic, threatened, and narrow range species on the one hand, and being severely challenged for biodiversity conservation on the other hand (Figure S1). All the rivers except Zhujiang originate in the Qinghai‐Tibet Plateau. With Changjiang being one of the longest rivers in the world, Huanghe carrying a large amount of suspended sediment load, Yarlung Zangbo the highest river in the world (in terms of altitude), and Nujiang and Lancang famous for their V‐shaped valleys (Liu, 2014), these river valleys together represent both typical and atypical habitats. The differences in ecosystem among these six LRVs provide opportunities for the flourishing of a variety of plant species. Furthermore, these six rivers play an important role in maintaining the floristic diversity of the Qinghai‐Tibet Plateau and surrounding areas and provide water and nutrients to foster the biodiversity in the respective river basins. The characteristics of these six rivers, such as length, drop, drainage area, and runoff volume, are listed in Table S1.1.

### 2.2 Database of species occurrence data

We georeferenced 124,927 occurrence points of seed plants distributed along these six large rivers with precise coordinate information, out of which 114,638 points were accessed from the Chinese Virtual Herbarium (http://www.cvh.ac.cn/) and the rest from Global Biodiversity Information Facility (GBIF.org [January 2, 2022] GBIF Occurrence Download https://doi.org/10.15468/dl.rwpdtt). In the process of determining the occurrence points mentioned above, we cleaned the distribution data obtained in the following manner. First, occurrence records without detailed locality information were excluded and the synonyms standardized according to *Catalogue of Life China* (Species 2000 China). Next, we georeferenced the remaining specimen records according to the China gazetteers using the SQL Server Management Studio (Microsoft SQL Server 2008) and manually fine‐tuned the results with the help of maps. Finally, we obtained the provincial distribution ranges of species from the Flora of China (http://www.efloras.org/) and rescreened them to delete the records that did not belong to the species range.

Endemism and endangered status have commonly been regarded as important criteria for identifying diversity hotspots (Huang et al., 2016; Myers et al., 2000). Therefore, we used the following four groups: endemic species, threatened species, nationally protected species, and all species. The concept of endemism refers to species restricted to a certain area (Anderson, 1994). The species endemic to China were identified based on the Catalogue of Life China 2021 Annual Checklist and *Diversity and Geographical Distributions of Chinese Endemic Seed Plants* (Huang et al., 2014). The species list used in this study is presented in Table S2, which lists the endemic, threatened, and nationally protected species and the names of the six large rivers. The categorization of threatened plants followed the Threatened Species List of China's Higher Plants (Qin et al., 2017) and the nationally protected plants were listed according to the List of Wild Fauna and Flora under State Key Protection (issued by Ministry of Forestry and Ministry of Agriculture in 1989).

### 2.3 PD analysis

For phylogenetic inference, we generated a species‐level phylogenetic tree using the “phylo.maker” function implemented in the R (version 4.0.2) package, V. PhyloMaker (Jin & Qian, 2019). The package uses the largest dated phylogeny for plants, GBOTB (Smith & Brown, 2018) as a backbone for vascular plants. A study of community phylogenetics based on a phylogeny produced in this way has been recently shown to be equivalent to that based on a species‐level phylogeny (Qian & Jin, 2021), paving the way for this approach to be used to reconstruct plant phylogenies to measure PD (Qian & Deng, 2021; Shrestha et al., 2021). We extracted the information about the root and basal nodes of the genera to generate a phylogenetic hypothesis based on the function *build*.*nodes*. 1. Finally, 14,448 species (accounting for 99.78% of all species) belonging to 2028 genera and 212 families were included in V. PhyloMaker (Table S2). We considered two PD metrics, namely PD (Faith, 1992) and phylogenetic endemism (PE) (Rosauer et al., 2009), calculated using Biodiverse V2.0 (Laffan et al., 2010). Using the same software, we also calculated WE to study differences in area of the species distribution (Crisp et al., 2001). We treated PD, PE, and WE collectively in spatial phylogenetics for convenience.

### 2.4 Distribution pattern of species richness and conservation hotspots

We used ArcGIS 10.6 (ESRI) to create a buffer zone extending 20 km on either side of each river. Next, we divided the map of China into a grid of 4204 cells with a resolution of 50 km × 50 km (approximately 2500 km^2^ per cell) to reduce sampling artifacts such as the occurrence of artificially empty grid cells and mapping errors (Crisp et al., 2001; Linder, 2001). Of these, only 350 cells were wholly or partially occupied by the buffer zones of the six LRVs. We followed a method similar to the one adopted in biodiversity surveys (Crisp et al., 2001; Linder, 2001; Morawetz & Raedig, 2007) and examined the conservation indicators to detect aggregates of grid cells with high numbers of threatened, nationally protected, endemic, and all species.

We employed two algorithms to detect the distribution patterns of the four plant groups, namely the “species richness algorithm” and “complementary algorithm” (Dobson et al., 1997; Prendergast et al., 1993). The species richness algorithm focuses on species richness (number) of the four plant groups in each grid cell. The complementary algorithm determines the minimum area needed to cover all the species (Chi et al., 2017; Dobson et al., 1997). The specific operational steps of the complementary algorithm are to first select the grid cell with the highest species richness and remove all other species that occur in this grid cell from the database, repeating the above operation by selecting the grid cell with the highest number of the remaining species and removing all the other species in this grid cell from the database. This process continues iteratively until all species are included in the selected grid cells (Dobson et al., 1997).

Using the above steps, we obtained the value of each grid cell for corresponding plant groups by applying the two algorithms and the value of PD, PE, and WE from spatial phylogenetics. Next, we standardized the value in each grid cell by calculating the ratio of the value in the grid cell to that of the cell with the highest value of the corresponding plant groups. We then selected the top 10% of the grid cells with the highest standardized values as hotspots for the respective plant groups of different algorithms. If multiple grid cells contained the same value, we selected all of them to avoid missing important cells. In the next step, we summed the different standardized values in each grid cell and sorted the sum in descending order and selected the top 10% grid cells with the highest sum as the final plant diversity hotspots. Finally, we used ArcGIS 10.6 to visualize the distribution patterns and hotspots presented in this study. We did two analyses for these six LRVs: an integrated analysis by treating all six LRVs together and separate analyses for each river valley. We did the latter analyses because of the large differences in species diversity and floristics among these six LRVs and to exhibit the specific features of biodiversity conservation in each river valley.

### 2.5 Conservation effectiveness and gap analyses

We performed conservation effectiveness and gap analyses based on the distribution pattern of hotspots and the distribution of national/provincial nature reserves. For this purpose, we constructed shape files for 464 national nature reserves (NNRs) and 785 provincial nature reserves (PNRs) based on relevant documents issued by the government until 2018. We then overlapped the nature reserve layer with the combined hotspots to generate maps of conservation effectiveness and gaps, by recognizing overlapped/nonoverlapped hotspot grid cells (Hou et al., 2010; Zhang et al., 2016). We analyzed the data for endemic, threatened, nationally protected, and all plant species to determine the conservation effectiveness and gaps.

### 2.6 Correlation between distribution patterns and species composition

To assess the congruence of different distribution patterns identified by different groups and algorithms, we conducted a pairwise correlation analysis on the distribution patterns of species richness and species complementarity of the four plant groups, as well as the spatial phylogenetics indices using the “corrplot” (version 0.84) package (Wei & Simko, 2017) in R (version 4.0.2). We constructed a matrix for the correlation analysis with the plant groups as columns and grid cell IDs as rows (Table S3). As empty cells are not permitted by the program, they were given the value “0.” The distribution pattern of all species based on the species richness algorithm did not contain any empty cells, and therefore, there were no cells with “0” for this group. The other groups, however, obviously with fewer species, did contain such cells. Since the spatial phylogenetics was based on the phylogenetic tree inferred using all the species, the distribution patterns of PD, PE, and WE covered all the grid cells, and therefore, the columns of PD, PE, and WE in the matrix contained values other than zero. On the other hand, since the complementary algorithm is aimed at selecting the minimum area required to cover all the species (Chi et al., 2017; Dobson et al., 1997), the distribution pattern under this algorithm only covered partial grid cells when compared to the distribution pattern of all species obtained for the species richness algorithm. Therefore, the columns associated with the complementary algorithm did contain multiple zero‐valued cells. Subsequently, we standardized and log‐transformed the values to approximate a normal distribution and used Pearson’s correlation to measure associations. We used the following conventional interpretations of the *r* value: |*r*| ≥ .8 for very strong correlation, .6 ≤ |*r*| < .8 for strong correlation, .4 ≤ |*r*| < .6 for moderate correlation, .2 ≤ |*r*| < .4 for weak correlation, and .0 ≤ |*r*| < .2 for very weak correlation or no correlation (Jain & Chetty, 2019).

In order to elucidate the species composition of different plant groups in the hotspots, NNRs, and PNRs in conservation effectiveness and gap analysis, we illustrated the relationships of different plant groups using the “circlize” (Version: 0.4.12) package (Gu et al., 2014) and “tidyverse” (Version: 1.3.1) package (Wickham et al., 2019). These figures present the proportion of the hotspot grid cells and different plant groups in the form of chord diagrams and circular bar plots. For visualizing the species composition, we considered the threatened species, endemic species with threatened species excluded, and the remaining species with threatened and endemic species excluded, separately, to avoid overlapping the different conservation priorities.

## 3 RESULTS

### 3.1 Distribution pattern of species richness and PD

We recorded a total of 14,481 plant species from the six LRVs, belonging to 2047 genera and 215 families (Table S2). Of these, 129 were gymnosperms and the remaining (14,352) were angiosperm species. Together, they accounted for 44.80% of the spermatophytes in all of China, with 51.39% of the gymnosperms and 44.74% of the angiosperms represented. About 50% of the species (7113) are endemic to China, belonging to 1085 genera and 159 families, accounting for 38.34% of all the endemic species.

**FIGURE 1:**
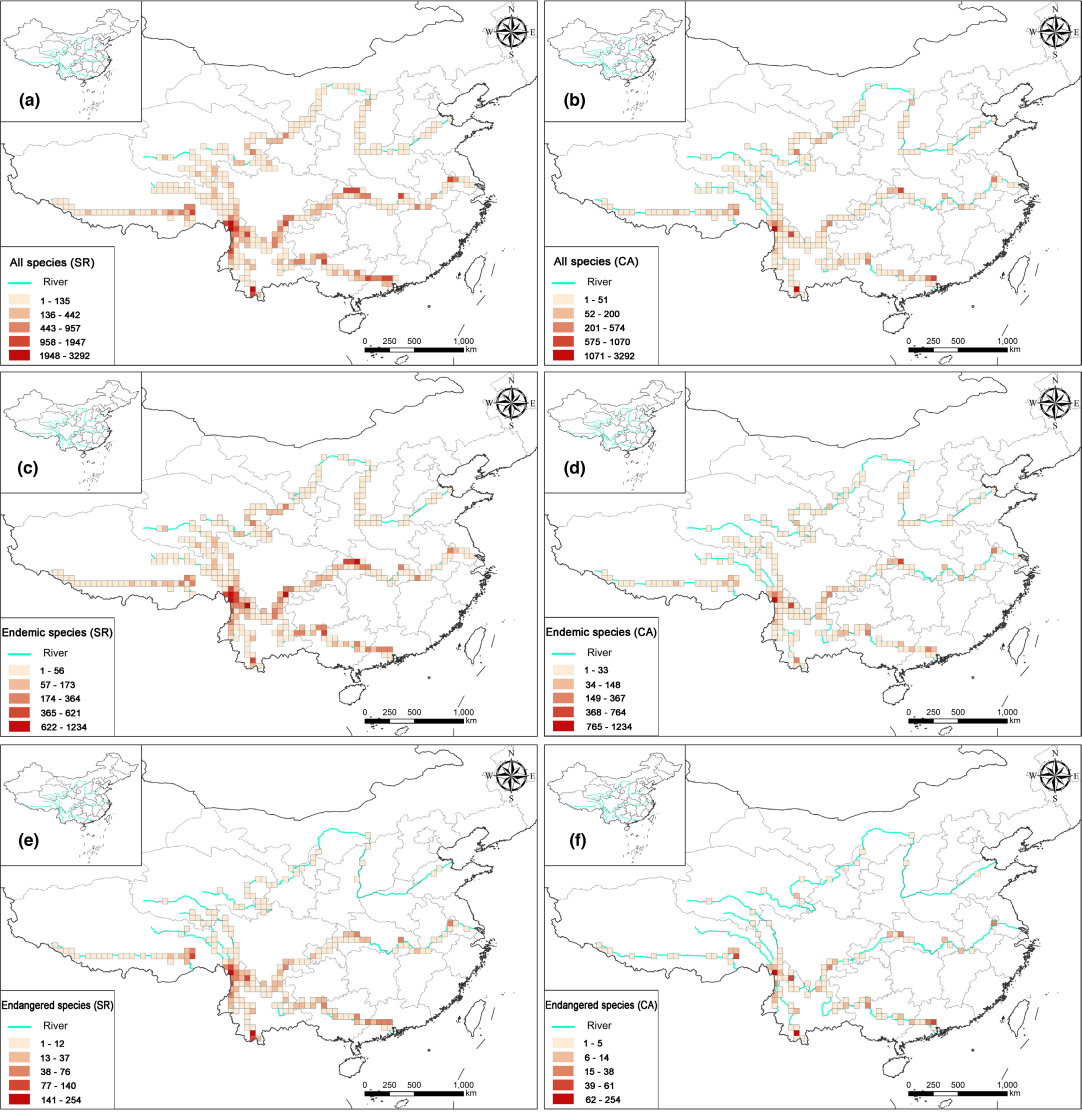
Species richness along the six Chinese large river valleys based on the integrated analysis. Grid cells are 50 km × 50 km. All the species: grid cells are determined by (a) the species richness (SR) algorithm and (b) the complementary algorithm; endemic species: grid cells determined by (c) the species richness (SR) algorithm and (d) the complementary algorithm (CA); and threatened species: grid cells are determined by (e) the species richness (SR) algorithm and (f) the complementary algorithm (CA)

Using the species richness algorithm, we determined that the Three Parallel Rivers region, the bend of the Yarlung Zangbo and the downstream part of the Lancang contained the highest richness in all the groups—endemic species, threatened species, nationally protected species, and all species (Figure 1 and Figure S2). The middle reaches of the Changjiang (the Bashan‐Wushan region) and the downstream part of the Zhujiang are characterized by greater richness of the endemic, threatened, and nationally protected species. The headwater region of Huanghe is characterized by a large number of grid cells with low species richness of the nationally protected species (Figure S2). Compared to the species richness algorithm, the complementary algorithm identified grid cells with high species richness mostly in the Three Parallel Rivers region, the middle reaches of the Changjiang (the Bashan‐Wushan region), and the downstream reaches of the Lancang and Zhujiang rivers (Figure 1). The distribution patterns based on the species richness algorithm are largely congruent among the endemic, threatened, nationally protected, and all species, differing only in species richness among the grid cells. Finally, the distribution pattern identified by the complementary algorithm was similar to that of the species richness algorithm, except that each group showed a more scattered distribution (Figure 1).

**FIGURE 2:**
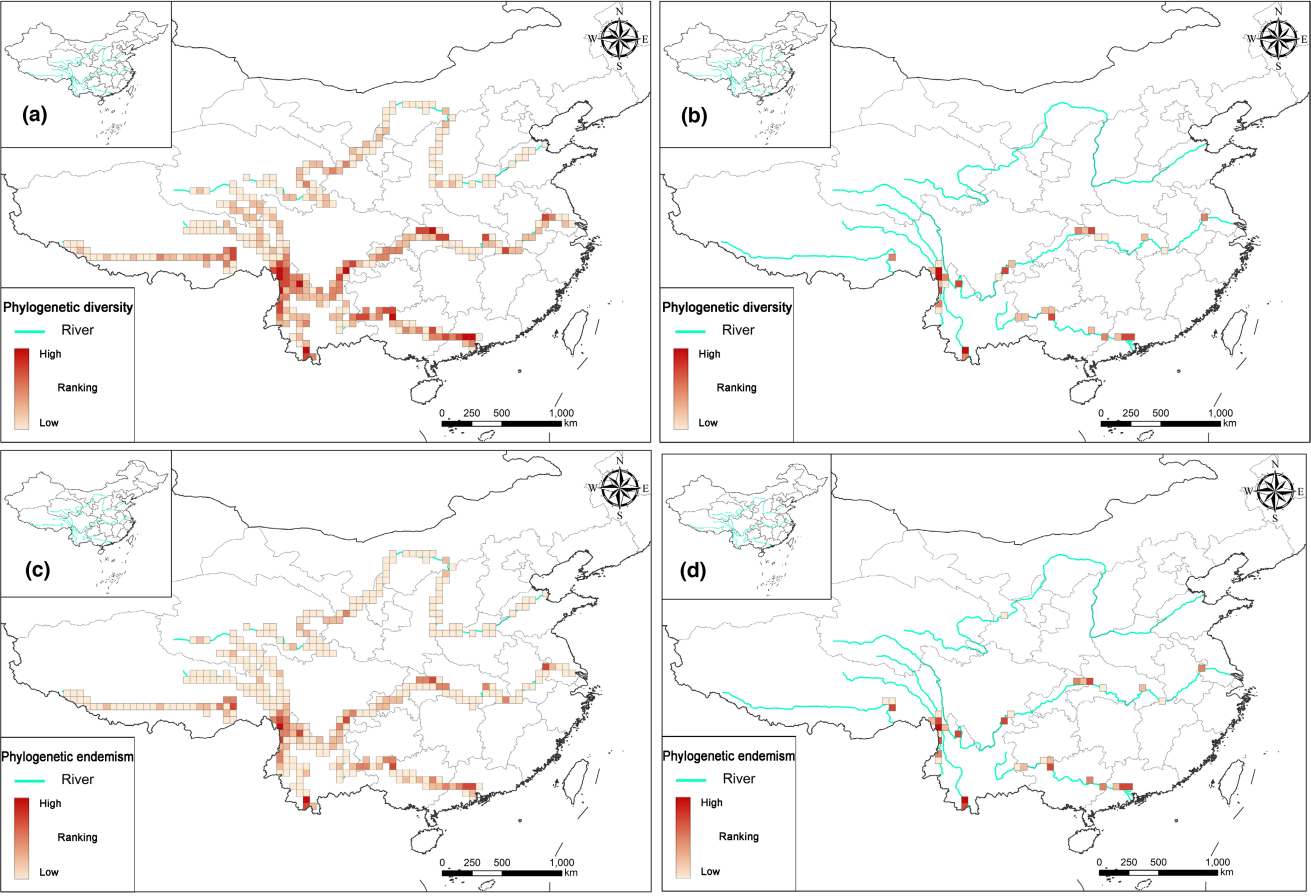
Distribution patterns of spatial phylogenetics along six river valleys based on the integrated analysis. Distribution patterns of (a) phylogenetic diversity (PD) and (c) phylogenetic endemism (PE); hotspot grid cells of (b) phylogenetic diversity (PD) and (d) phylogenetic endemism (PE)

**FIGURE 3:**
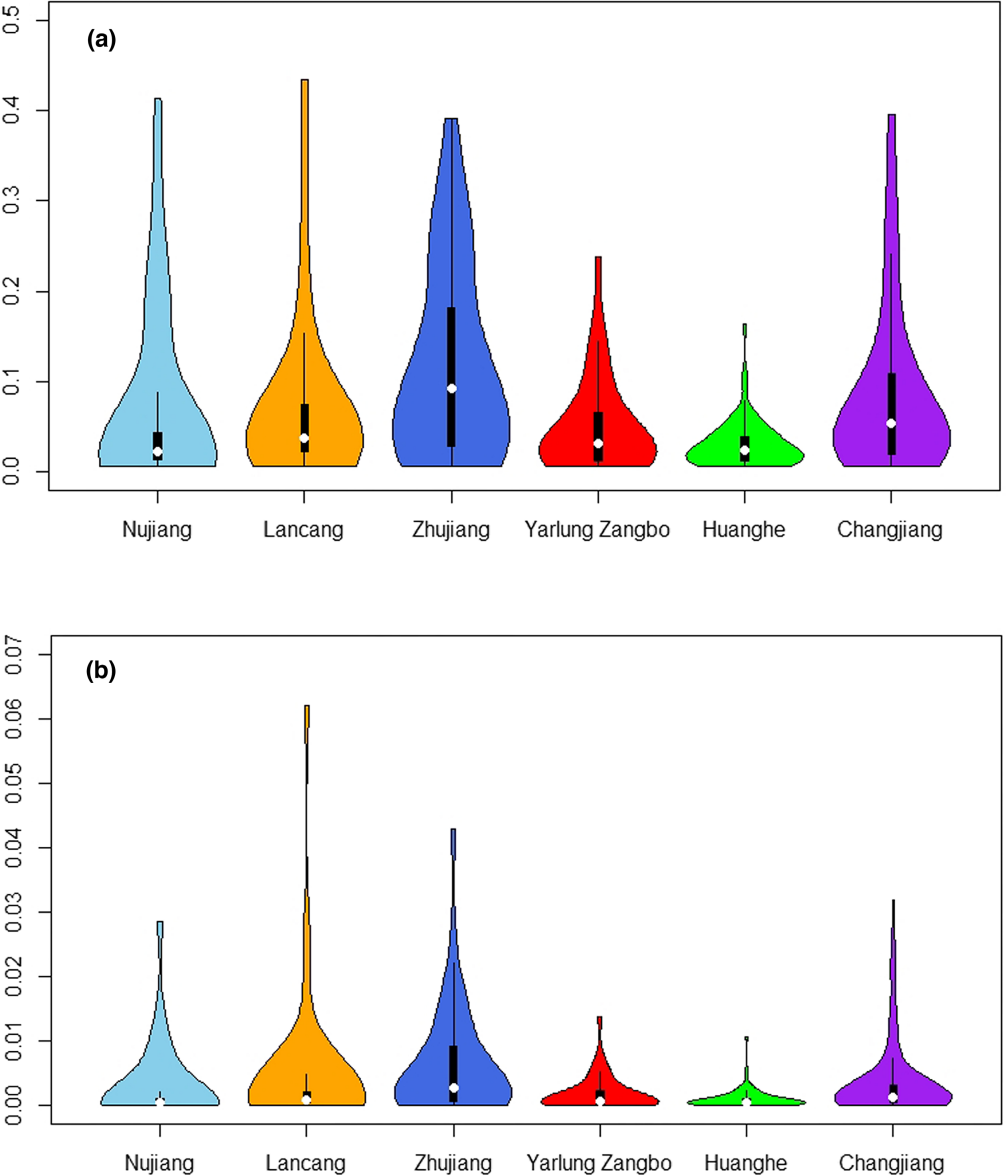
Violin plots of (a) phylogenetic diversity (PD) among six river valleys, and (b) phylogenetic endemism (PE) among the six Chinese large river valleys chosen in this study

According to the spatial phylogenetics, the hotspot distribution patterns of PD, PE, and WE were largely congruent with each other (Figure 2 and Figure S3). Grid cells with high values of PD and PE were mostly confined to the Three Parallel Rivers region, the middle reaches of the Changjiang (the Bashan‐Wushan region), and downstream of the Lancang and Zhujiang rivers (Figure 2), whereas the distribution pattern of WE was somewhat scattered (Figure S3). The grid cells with high WE values were much more scattered, making it difficult to identify clustered grid cells with high WE value (Figure S3). Although the PD and PE values of the valleys along the Changjiang and Zhujiang rivers were higher compared to those along the other valleys (Figure 3), the Lancang had the highest values of PD, PE, and WE, overall, while the Huanghe and Yarlung Zangbo rivers had the lowest (Table S4). Finally, there was more variation in the values for PD than for PE or WE, among the six LRVs (Figure 3 and Figure S4).

### 3.2 Correlation between distribution patterns

**FIGURE 4:**
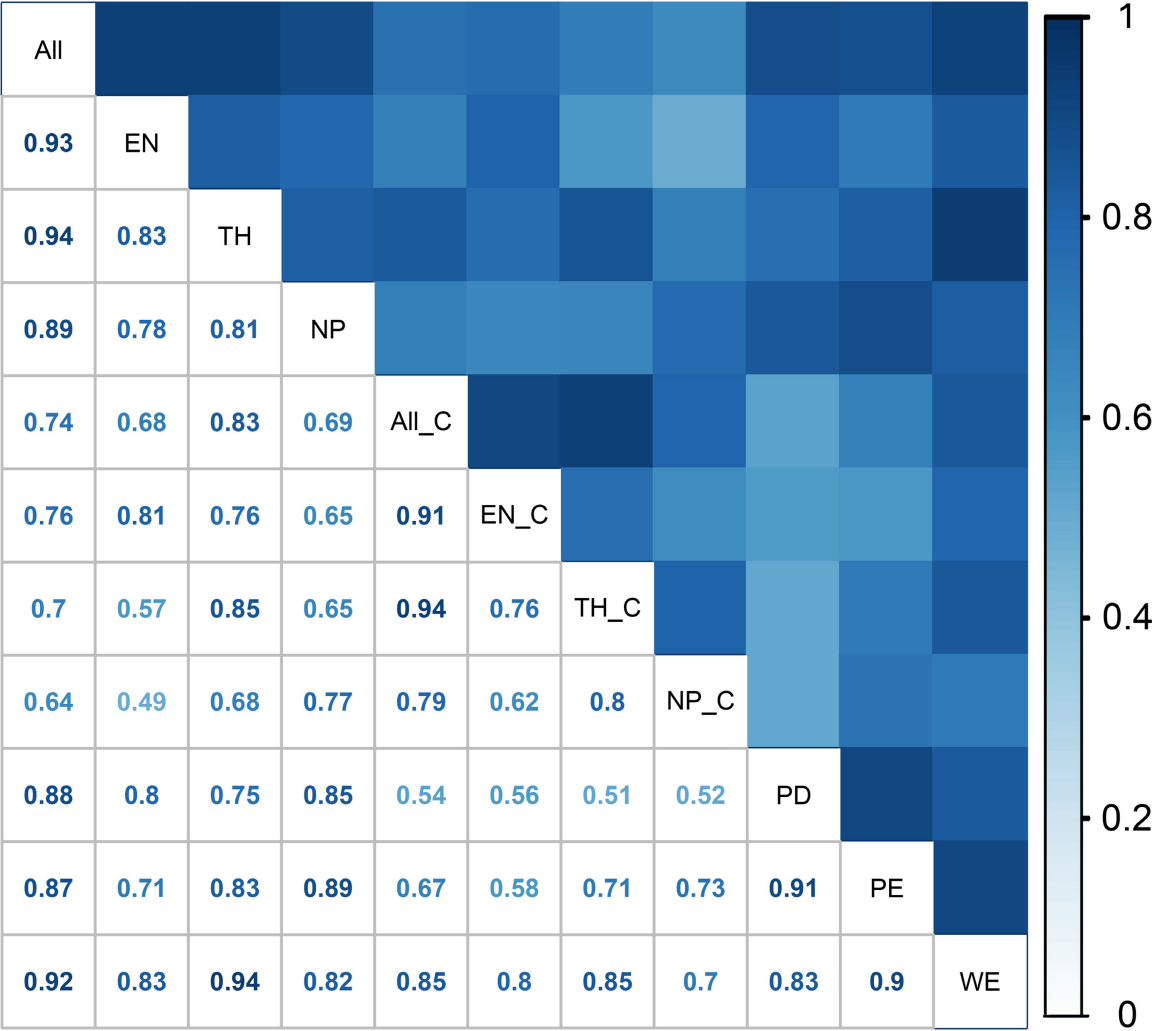
Pairwise correlations between species distribution patterns and spatial phylogenetics: all the species (All), endemic species (EN), threatened species (TH), and nationally protected species (NP) according to the species richness algorithm; all the species (All_C), endemic species (EN_C), threatened species (TH_C), and nationally protected species (NP_C) according to the complementary algorithm; and phylogenetic diversity (PD), phylogenetic endemism (PE), and weighted endemism (WE) according to spatial phylogenetics

Figure 4 shows the correlation coefficient for the pairwise comparisons among the various groups of plant species under each of the two algorithms as well as PD, PE, and WE under spatial phylogenetics. As can be seen, very strong correlations (*r* = .82–.95, *p* < .01) were seen among the endemic, threatened, nationally protected, and all species when the species richness algorithm was used, and also between the endemic and all species, threatened and all species, and threatened and nationally protected species, when the complementary algorithm was used. Very strong pairwise correlations were also seen among PD, PE, and WE, for endemic species and threatened species between the two algorithms and for the threatened species and all species between two algorithms. Most other correlations were in the range of moderate to strong (Figure 4).

Correlation analysis also showed that distribution patterns of the algorithms presented apparent mismatches (Figure S5). In general, there were very strong correlations among different plant groups or indicators within each algorithm, especially along the Nujiang river. For the species richness algorithm and complementary algorithm, there were strong to very strong correlation of the distribution patterns along the valleys of Changjiang, Huanghe, Nujiang, and Zhujiang rivers (Figure S5a,b,d,f) and there were weak to strong correlations along the valleys of Lancang and Yarlung Zangbo rivers (Figure S5c,e). While the correlations were strong to very strong between the distribution patterns of the species richness algorithm and spatial phylogenetics along five LRVs, they were weak to strong along the Yarlung Zangbo valley (Figure S5). There were strong to very strong correlations between the distribution patterns derived from the complementary algorithm and spatial phylogenetics in the six LRVs, except the distribution pattern of nationally protected species according to the complementary algorithm and spatial phylogenetics, which presented moderate correlations along valleys of Huanghe and Yarlung Zangbo (Figure S5).

### 3.3 Distribution pattern of diversity hotspots

The distribution patterns of hotspots identified by the species richness algorithm and the complementary algorithm were similar when each river was considered individually (Figure S6). More hotspots were detected by the complementary algorithm along the Huanghe and Yarlung Zangbo rivers when compared to the species richness algorithm, with a few hotspot grid cells in the Three Parallel Rivers region as well (Figure S6). However, more hotspots were identified in the headwaters of Huanghe when each river was considered individually, compared to when the six LRVs were integrated in the analysis, regardless of the algorithm (Figure S6). Although the Zhujiang and Changjiang rivers had a higher mean species richness on average (Figure S7), the Nujiang and Lancang rivers had individual regions with higher values of species richness (Table S4). The hotspots detected with respect to PD, PE, and WE were more similar to those from the species richness algorithm than from the complementary algorithm (Figure S6).

**FIGURE 5:**
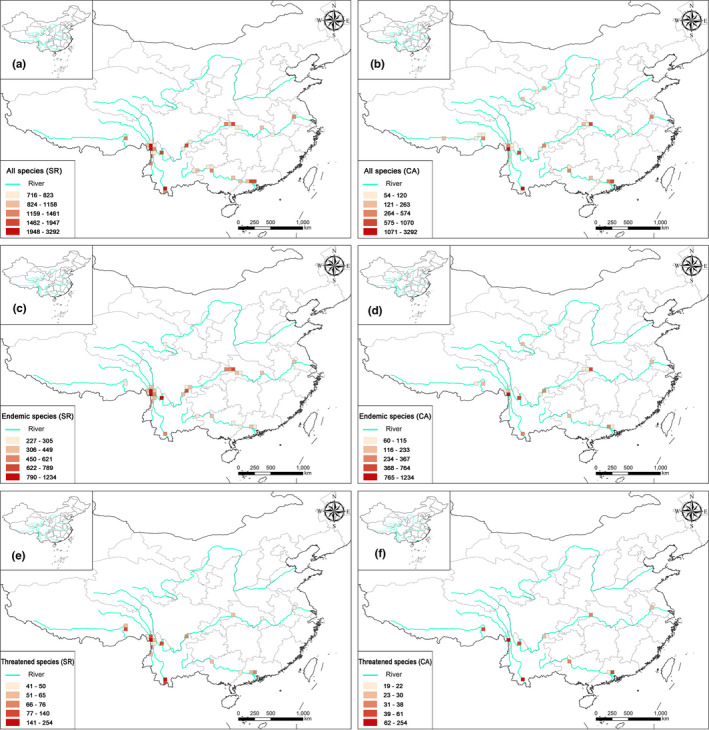
Hotspot grid cells determined by the species richness (SR) algorithm: (a) all the species, (c) endemic species, and (e) threatened species. Hotspot grid cells determined by the complementary algorithm (CA) based on integrated analyses: (b) all the species, (d) endemic species, and (f) threatened species

**FIGURE 6:**
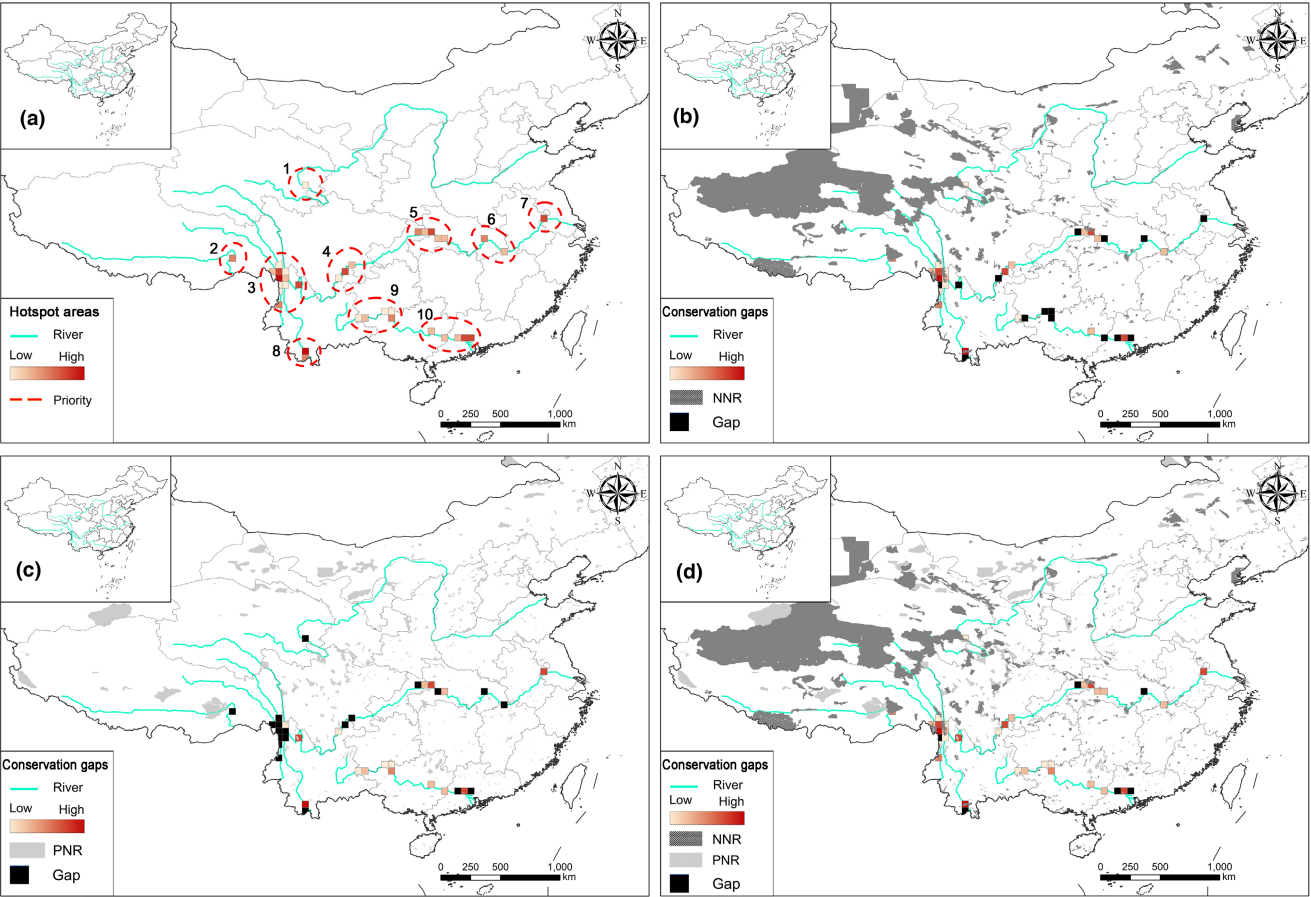
Hotspots and conservation gaps along the six large rivers in China. (a) Ten diversity hotspots detected in this study. (b) Conservation effectiveness of national nature reserves (NNRs), with conservation gaps shown in black. (c) Conservation effectiveness of provincial nature reserves (PNRs), with conservation gaps shown in black. (d) Conservation effectiveness of NNRs and PNRs with conservation gaps shown in black

The hotspots of all species and threatened species were mostly confined to the Three Parallel Rivers region and lower reaches of the Lancang (Figure 5a,b,e,f). For endemic species, the grid cells with higher species richness were in the Three Parallel Rivers region, lower reaches of the Lancang, and the Bashan‐Wushan region (Figure 5c,d). Hotspots of nationally protected species were identified in the Three Parallel Rivers region, downstream of the Lancang, and the estuary of the Zhujiang (Figure S2b,d). A limited number of nationally protected plants were identified along the Huanghe and Yarlung Zangbo rivers. Finally, the top 10% grid cells with higher species richness, species complementary, and PD were identified as conservation hotspots. There were ten hotspots covering 36 cells, namely (1) the headwater region of the Huanghe, (2) the bend of the Yarlung Zangbo, (3) the Three Parallel Rivers region, (4) the eastern Hengduan Mountain region, (5) the Bashan‐Wushan region, (6 and 7) the middle and lower reaches of the Changjiang, (8) the downstream part of the Lancang, and (9) the upstream and (10) the downstream regions of the Zhujiang (Figure 6a). The 36 hotspot grid cells contained a total of 12,081 species, with 5778 endemic, 951 threatened, and 64 nationally protected species, accounting for 83.4%, 81%, 85.6%, and 90% of the corresponding plant groups of the six LRVs, respectively (Table S1.2).

### 3.4 Conservation effectiveness and gaps

Conservation effectiveness analysis indicated that 8 of 10 hotspot areas were covered partially by the NNR networks. However, only 58% of the area of the hotspots is protected by the current NNR networks (Figure 6b). These hotspot areas covered by the NNR networks contain 84% of all species, 81% of endemic species, and 82% of threatened species, respectively, among the corresponding hotspots (Figure 6b and Table S1.2). There were hotspot grid cells in the lower reaches of the Changjiang and the upper reaches of the Zhujiang within the PNR conservation networks as well (Figure 6c). The PNR networks covered 44% of the hotspot area, harboring 8373 species (69%), and 64% of endemic species as well as 62% of threatened species, among the corresponding hotspots (Figure 6c and Table S1.2). Together, the conservation networks of NNR and PNR covered 83% of the area of hotspots, including 96% of all species, 5553 species endemic to China, and 904 threatened species (Figure 6d and Table S1.2).

Forty‐four percent of the area of hotspots was identified as conservation gaps in the NNR networks, containing a total of 7517 species (62%), of which 57% are endemic and 51% are threatened in the hotspot grid cells, respectively (Table S1.2). Furthermore, there were an aggregate of 2063 species (17%), with 19% of endemic and 18% of threatened among the hotspot grid cells, respectively. The conservation gaps in NNRs and PNRs comprised 17% of the area of hotspots, containing 36% of all species, 25% of endemic species, and 28% of threatened species (Table S1.2). There were also 565 species (225 endemic and 47 threatened) that made up a small percentage of the corresponding plant groups in the hotspot grid cells that occurred outside the conservation networks of NNRs and PNRs. Apparent conservation gaps among the NNRs were found scattered mostly along the Zhujiang and Changjiang rivers (Figure 6b). Upon overlaying them on PNR networks, however, the conservation gaps were found to be distributed along the Zhujiang, Changjiang, Nujiang, and Lancang rivers, with most of them being confined to the Bashan‐Wushan region, the lower reaches of the Zhujiang, the Three Parallel Rivers region, and lower reaches of the Lancang (Figure 6d).

### 3.5 Species composition in hotspots and current conservation networks

There were 225 grid cells that fell within NRs (NNRs and PNRs), accounting for 64% of the land area in six LRVs, in which 13,500 species naturally occur (1034 threatened species and 5998 endemic species excluding threatened species; Table 1). Statistics on species composition in the hotspots, NNRs, and PNRs indicate that the hotpots identified in this study included more species (12,081 species including 951 threatened and 5778 endemic species) compared to those in NNRs or PNRs (Figure S8 and Table S1.2). The grid cells covered by NNRs accounted for 40% of the area and contained 11,854 species (including 894 threatened and 5554 endemic). There were 140 grid cells (occupying 40% of the area) under PNRs, which included 10,468 species (730 threatened and 4799 endemic). NNRs accounted for more threatened and endemic species than did PNRs, although they occupied the same number of grid cells (Table 1 and Table S1.2). Together, there were 225 grid cells covered by NRs, accounting for 64% of the land area, with 13,500 species (1034 threatened and 6583 endemic species excluding threatened species).

**TABLE 1:** The species composition of threatened species (TH), endemic species with threatened species excluded (EN), and the remaining species with threatened and endemic species excluded (REMA) in the six LRVs, hotspots, conservation networks, conservation effectiveness, and gaps

Taxonomic groups	Species number of six LRVs	Species number of hotspots	Species number of NNRs	Species number of PNRs	Species number of NNRs and PNRs	Conservation effectiveness of NNRs	Conservation effectiveness of PNRs	Conservation effectiveness of NNRs and PNRs	Conservation gaps of NNRs	Conservation gaps of PNRs	Conservation gaps of NNRs and PNRs
TH	1111	951	894	730	1034	783	594	904	483	657	271
EN	6474	5252	5072	4396	5998	4274	3404	5059	3030	3814	1311
REMA	6896	5878	5888	5342	6468	5149	4375	5645	4004	4824	2751
Total species	14,481	12,081	11,854	10,468	13,500	10,206	8373	11,608	7517	9295	4333
Grid cells	349	36	140	140	225	21	16	30	15	20	6

The hotspot grid cells covered by NNRs and PNRs together contained 11,608 species (5963 endemic species and 904 threatened), with NNRs containing considerably more than PNRs, highlighting the differences in conservation effectiveness between them (Figure S9 and Table 1). In another sign of the conservation measures in NNRs being more effective, there were 3513 endemic species and 483 threatened species in gaps within NNRs and 4471 endemic species and 657 threatened species in the gaps within PNRs. The conservation gaps of NNRs and PNRs included six hotspot grid cells, accounting for 1.7% of the area of six LRVs, but containing 29.9% of all species, which included 24.4% of the threatened species and 20.3% of the endemic species with the threatened species excluded. Further details may be found in Table 1 (Figure S9).

## 4 DISCUSSION

### 4.1 Biodiversity conservation priority planning in LRVs

Large river valleys play a vital role in conserving biodiversity as they harbor large and important natural resources (Chawla et al., 2008). The 40‐km buffering zones along these LRVs, while covering only about 3.11% of the land area of China, contained about 40% of the seed plants of the country. The hotspots along six LRVs contain the bulk of the flora and are consequently vital for biodiversity conservation and sustainability. These hotspots are characterized by higher proportions of threatened and nationally protected species (Table S1.3). However, there was a marginally smaller proportion of endemic species in the hotspots (47.83%) when compared to those in the study area (49.12%), perhaps due to the apparent mismatches in the distribution patterns of endemic species and other plant groups (Figure 4 and Figure S5). To the extent that these mismatches are responsible for the differences, they need to be considered while prioritizing among the hotspots for biodiversity conservation. It is worth noting that the three hotspots in Southwest China are adjacent to India, Myanmar, and Laos, and the three rivers corresponding to the hotspots flow into these countries (Figure 6). We propose that planning the prioritization of the conservation of plant diversity in the river valleys should be done in consultation with all the neighboring countries. This approach would better respond to China's Belt and Road Initiative, an unprecedented global development program in pursuit of economic growth consistent with sustainable development goals based on environmentally friendly approaches (Ansari et al., 2021).

The hotspots identified by the species richness algorithm tended to be more clustered, compared to those detected by the complementary algorithm (Figure 5 and Figure S6). Since the complementary algorithm identified areas with low species richness but high species irreplaceability, the results shed new light on understanding conservation priorities. For example, only one grid cell each as a hotspot in the headwater area of the Huanghe and the bend of the Yarlung Zangbo was identified as a priority area using the species richness algorithmss (Figure 5 and Figure S6a), while three and five grid cells, respectively, were identified as hotspots along these two river valleys using the complementary algorithm (Figure 5 and Figure S6c). There were mismatches between the distribution patterns of endemic species derived from different algorithms, as in the priority areas for threatened species and nationally protected species (Figure 4 and Figure S5). In addition, when the six LRVs were treated individually, there were remarkable mismatches in the distribution patterns in the valleys of Lancang, Yarlung Zangbo, and Zhujiang rivers between the endemic species according to the species richness algorithm and threatened species derived from the complementary algorithm, as well as endemic species accord to the former algorithm and nationally protected species derived from the latter algorithm (Figure S5). Therefore, both species richness and irreplaceability need to be considered for improving conservation effectiveness (Chen et al., 2002; Xue et al., 2021; Yang et al., 2021). Furthermore, while the species richness algorithm, complementary algorithm, and spatial phylogenetics identified many consistent hotspot areas, it appears that the complementary algorithm and the phylogenetic information are better at locating the grid cells with low species richness but significant conservation value.

### 4.2 Optimizing conservation networks along LRVs

The current conservation networks appear to be relatively effective in biodiversity conservation (Ren et al., 2015). Our results have shown that high proportions of the plant groups are protected by NNRs or PNRs, especially the endemic, threatened, and nationally protected species. However, conservation gap identified in this study also contained significant proportions of endemic, threatened, and nationally protected species (Table 1 and Table S1.2). Moreover, there were more grid cells in the conservation gaps than were protected in NNRs (Table 1). This implies that the layout of current conservation networks is not sufficient and should be optimized based on more relevant conservation indicators, especially along the LRVs.

Conservation efforts in the gap areas need to be strengthened, including optimizing the layout of conservation networks and establishing new nature reserves. For example, 10 grid cells, accounting for 44.44% of the hotspots, most of them along Changjiang and Zhujiang rivers, are considered conservation gaps by the NNRs. Therefore, the remaining conservation gaps, such as those in the Bashan‐Wushan area, the middle and downstream reaches of the Changjiang, the upper and downstream reaches of the Zhujiang, the Three Parallel Rivers region, and downstream reaches of the Lancang, are in an urgent need of protection to further strengthen the NNR networks. The PNRs provide a suitable complement to the NNRs (Figure 6). Indeed, many of the hotspots identified in this study, such as the ones in the lower reaches of the Changjiang (Figure 6) and the upstream region of the Zhujiang (Figure 6b), are outside the NNR networks, but within PNR networks (Figure 6c). However, the distribution of PNRs is rather uneven as they are confined mostly to the Zhujiang and Changjiang rivers, and absent along the Nujiang and Lancang rivers. In general, no NNR or PNR networks exist that focus specifically on the LRVs. Therefore, there is urgent need to optimize the distributions of NNRs and PNRs, with more attention to the conservation in the LRVs.

Generally, nature reserves in China do not extend across administrative boundaries (Su et al., 2019; Xu et al., 2017). The effect of this is more pronounced in LRVs, where hotspots such as the Three Parallel Rivers region, the eastern Hengduan Mountain region, the Bashan‐Wushan region, the lower reaches of the Changjiang, and the upper and lower reaches of the Zhujiang (Figure 6b–d) are all shared by different provinces. The barriers of administrative boundaries need to be overcome during the design and construction of nature reserves in the future. More effective transregional conservation networks are also recommended to reduce serious problems in conservation, such as low habitat connectivity and high levels of habitat isolation.

### 4.3 Specific conservation planning and policies for prioritizing conservation in LRVs

Although different algorithms and multiple plant groups have been employed during the identification of hotspots in the six LRVs in this study, the strength of the correlations varied among algorithms, plant groups, and different LRVs (Figure 4 and Figure S5). Therefore, more attention needs to be paid to the distribution patterns of different plant groups with respect to conservation. Correlation analysis also showed that there was remarkable incongruence between the biodiversity hotspots derived from different algorithms and plant groups. In addition, the distribution patterns of different plant groups were also incongruent among the LRVs. For example, nationally protected species presented apparent mismatches with other plant groups regardless of whether these six LRVs were treated in an integrated manner or in separate analyses, especially with respect to the distribution patterns of nationally protected species in the river valleys of Changjiang, Huanghe, and Yarlung Zangbo (Figure S5). Therefore, more specific conservation planning should be considered for achieving effective protection, focusing on the incongruence in the distribution patterns of different taxonomic groups, PD, and river valleys.

Hotspots identified in this study are largely consistent with those from previous studies, with the most number of hotspot grid cells being confined to the heartland of the country, in particular, the southwestern part of China, in the upper and middle reaches of these six large rivers (Figure 6), where factors such as economic underdevelopment have resulted in a high dependence on natural resources (Hou et al., 2010; Yu et al., 2014). Therefore, most hotspots encounter the most glaring contradictions between economic development and ecological and environmental protection (Huang & Cai, 2006; Yu et al., 2014; Zhang et al., 2001). Therefore, master planning at the national level needs to balance economic development on one hand and conservation on the other hand. In addition, it is also necessary to promote public consciousness regarding the significance of conserving biodiversity and provide basic education about the law, especially pertaining to the rights of the ethnic minorities in the affected regions.

The six LRVs analyzed in this study are characterized by high species richness, and therefore, attention needs to be paid with respect to making conservation a priority. Apart from mitigating habitat loss, LRVs also provide important refuge and shelter to various species during extreme climate oscillations and are important for the development of flora at the periphery of the river valleys under stable climatic conditions (Holestova & Douda, 2022). Our study presents the distribution pattern of species richness along six LRVs in China, reveals their significance in protecting and sustaining biodiversity, and proposes guidelines for their conservation. More in‐depth studies focusing on biodiversity and conservation priorities are required to understand the role of river valleys in speciation, vicariance, differentiation, and adaptive evolution, to ensure the health of biodiversity in China.

## AUTHOR CONTRIBUTIONS

**Xudong Yang:** Data curation (supporting); formal analysis (equal); methodology (equal); validation (equal); visualization (equal); writing – original draft (equal). **Fei Qin:** Validation (equal); visualization (equal); writing – review and editing (equal). **Tiantian Xue:** Methodology (equal); validation (equal); visualization (equal). **Changying Xia:** Writing – review and editing (supporting). **Sudhindra R. Gadagkar:** Validation (equal); writing – review and editing (lead). **Shengxiang Yu:** Conceptualization (lead); data curation (lead); formal analysis (equal); funding acquisition (lead); investigation (lead); methodology (equal); project administration (lead); supervision (lead); visualization (equal); writing – original draft (equal).

## CONFLICT OF INTEREST

The authors declare no competing interests.

## Supporting information

Fig S1‐S9

Table S1

Table S2

Table S3

Table S4

## Data Availability

Data used in this study are stored in Dryad (https://doi.org/10.5061/dryad.jq2bvq8bt).
