# Supplementary material for: Insights into plant biodiversity conservation in large river valleys in China: A spatial analysis of species and phylogenetic diversity
Source: Ecol Evol. 2022 May 19;12(5):e8940. doi: 10.1002/ece3.8940 (PMC9120211; doi:10.1002/ece3.8940)
Supplement: Supplementary file 1 — Fig S1‐S9 [file ECE3-12-e8940-s003.doc]

**Supporting information – Appendix 3**

**Figure S1.** Large valleys of the following six rivers in China. (a) Huanghe River in Wuhai, Inner Mongolia. (b) Changjiang River in Diqing, Yunnan. (c) Lancang River in Jinghong, Yunnan. (d) Nujiang River in Gongshan, Yunnan. (e) Zhujiang River in Xingyi, Guizhou. (f) Yarlung Zangbo River in Shigatse, Xizang.


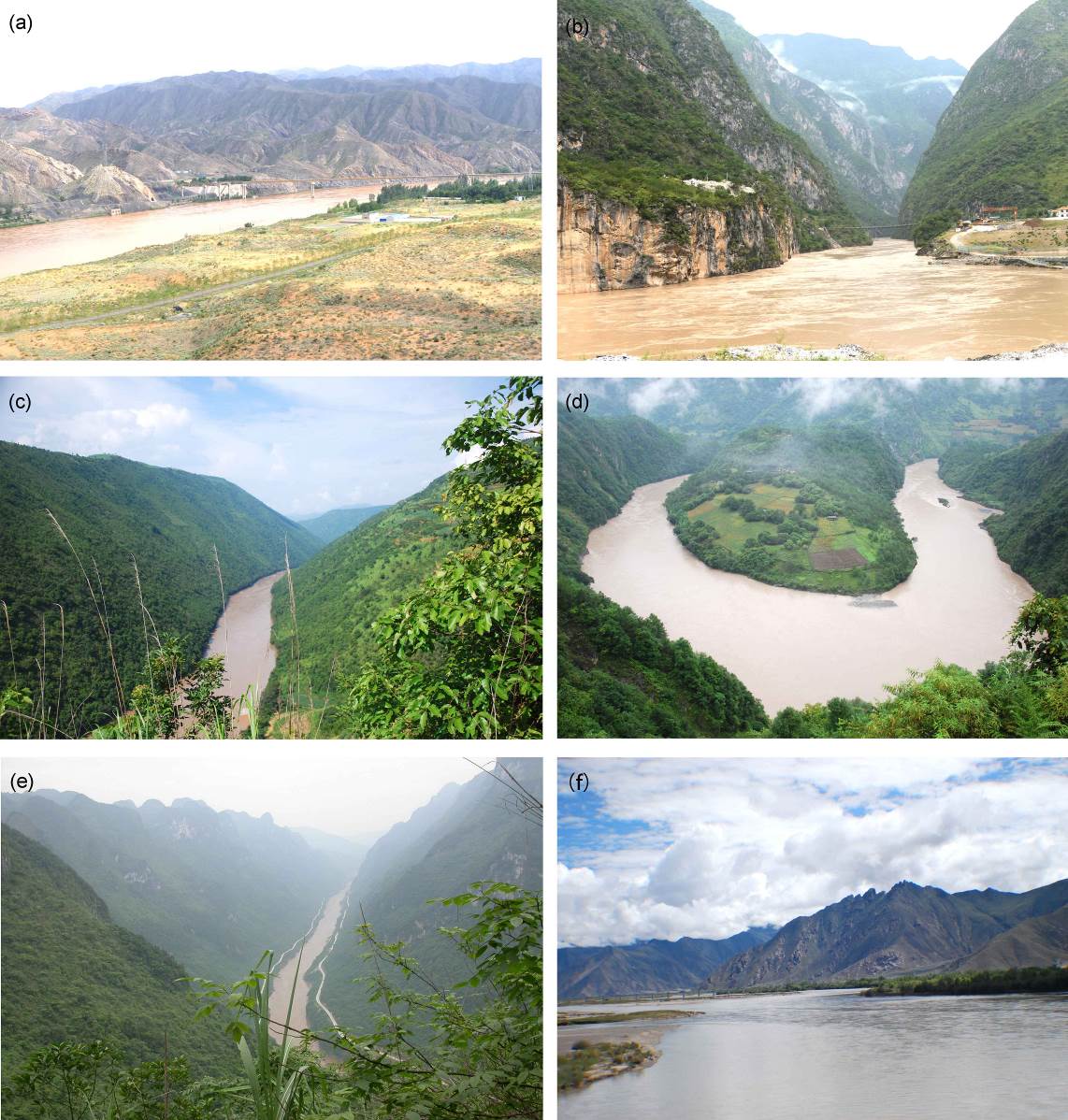


**Figure S2.** Species richness of nationally protected (NP) species. Grid cells are determined by: (a) the species richness (SR) algorithm; (b) the species richness algorithm with the top 10% species richness; (c) the complementary algorithm (CA); (d) the complementary algorithm with the top 10% species richness.

**
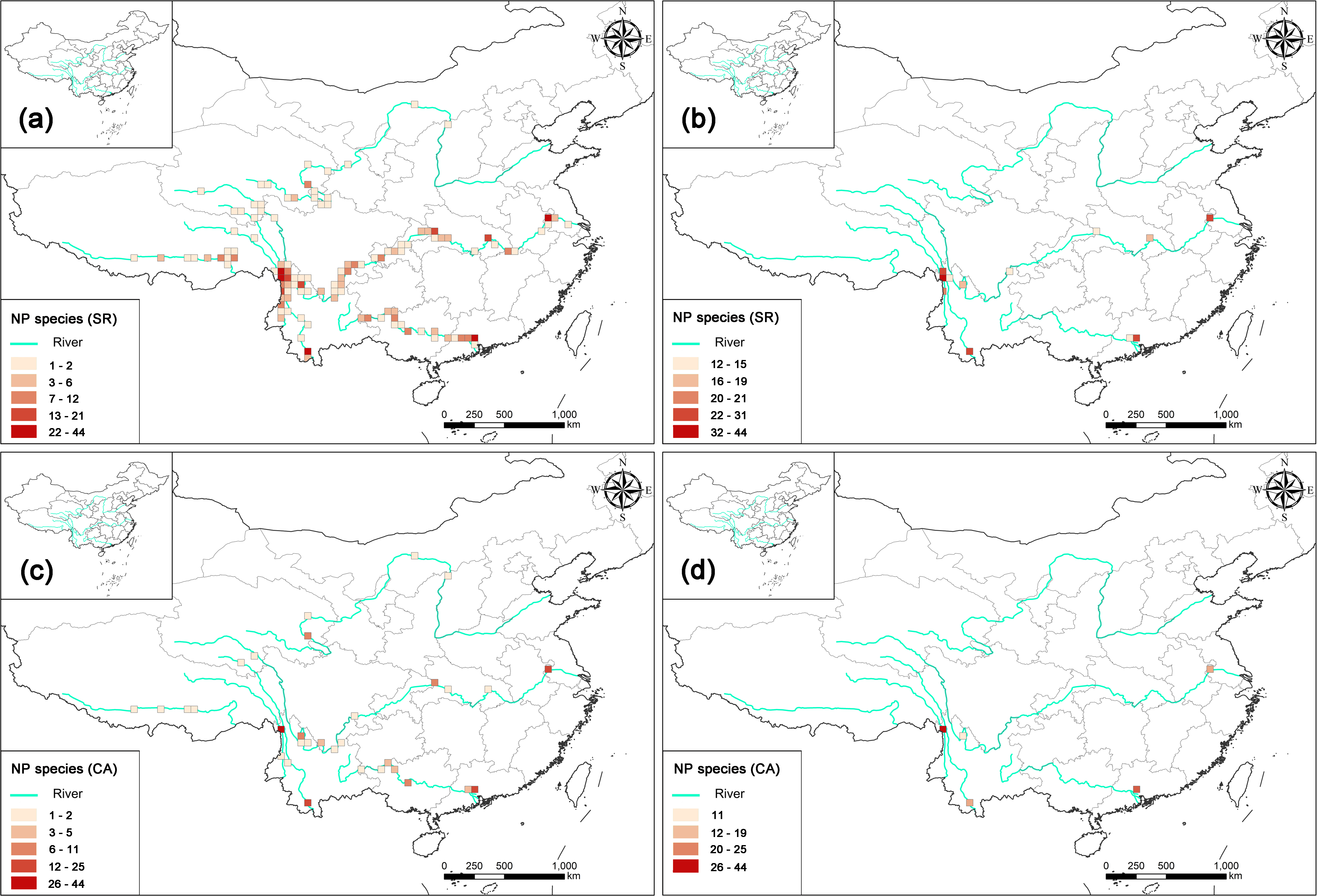
**

**Figure S3.** Spatial distribution patterns of weighted endemism (WE) along six river valleys.

**
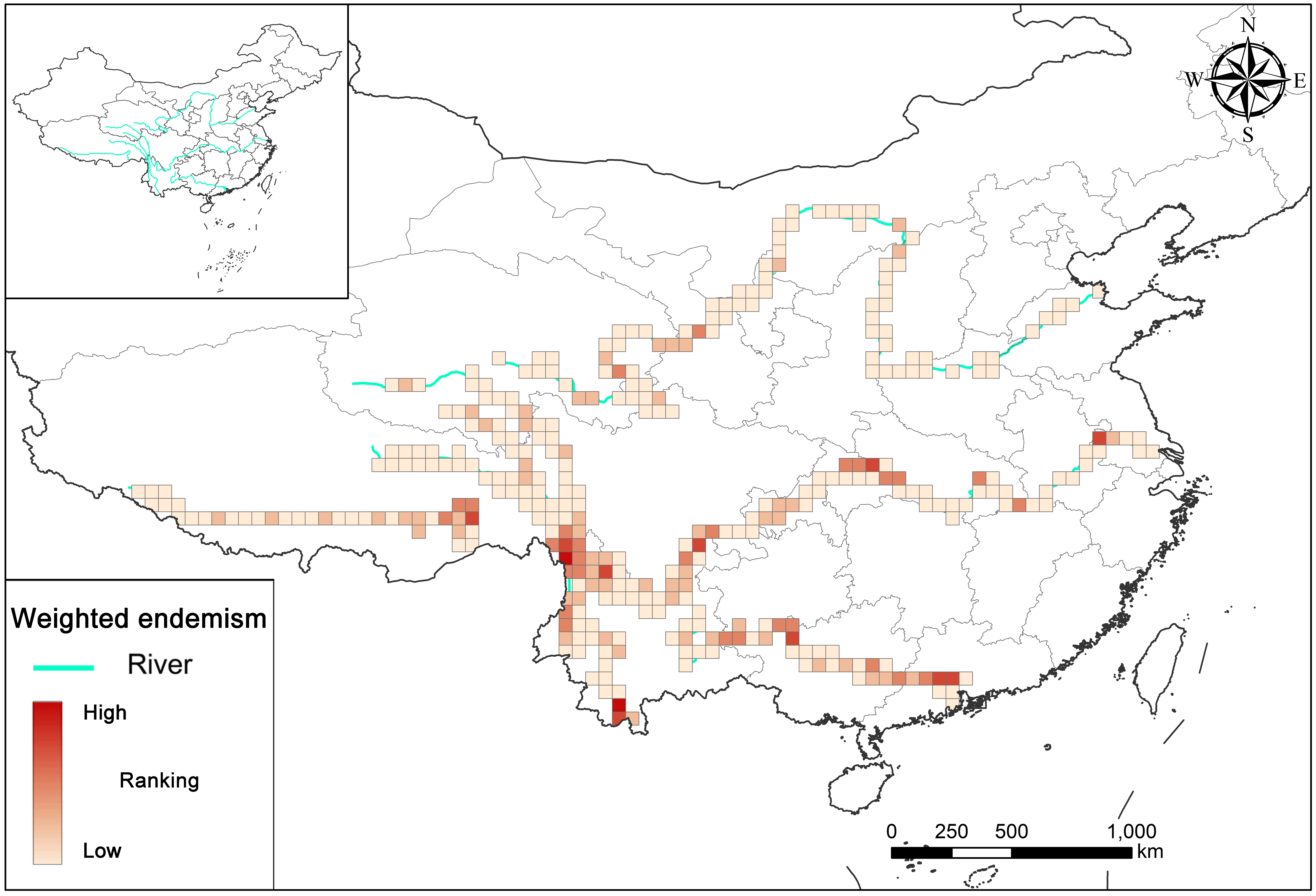
**

**Figure S4.** Violin plots of weighted endemism (WE) among six river valleys.

**
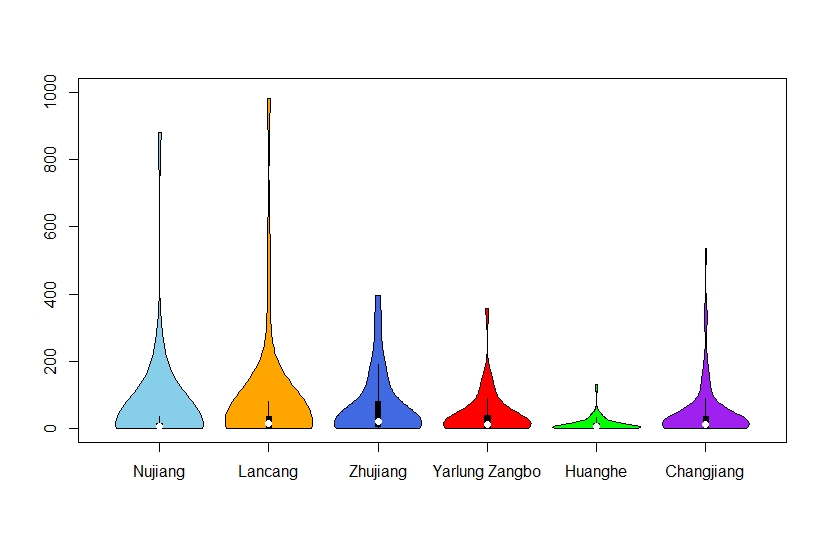
**

**Figure S5.** Pairwise correlations between species distribution patterns and spatial phylogenetics in (a) Changjiang, (b) Huanghe, (c) Lancang, (d) Nujiang, (e) Yarlung Zangbo, (f) Zhujiang rivers, respectively: all the species (All), endemic species (EN), threatened species (TH) and nationally protected species (NP) according to the species richness algorithm; all the species (All_C), endemic species (EN_C), threatened species (TH_C) and nationally protected species (NP_C) according to the complementary algorithm; phylogenetic diversity (PD), phylogenetic endemism (PE) and weighted endemism (WE) according to spatial phylogenetics.

**
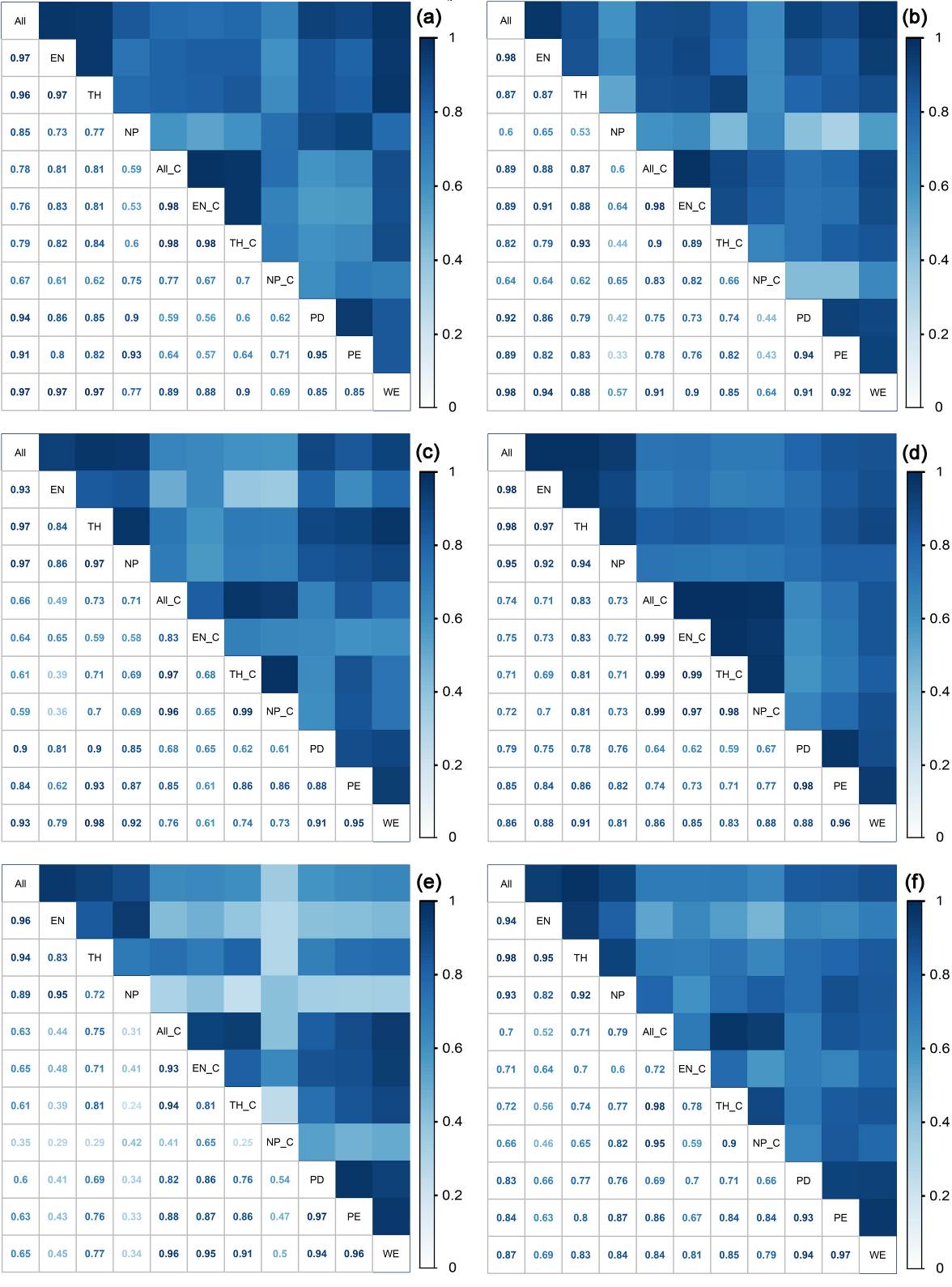
**

**Figure S6.** The hotpots of biodiversity along their valleys based on different criteria. (a) Hotspots of species richness according to the species richness algorithm by considering all six rivers based on the integrated analyses. (b) Hotspots of species richness according to the species richness algorithm by considering the six rivers individually. (c) Hotspots of species richness according to the complementary algorithm by considering all six rivers based on the integrated analyses. (d) Hotspots of species richness according to the complementary algorithm by considering the six rivers individually. (e) Distribution of hotspots of PD along each river, considering all six rivers based on the integrated analyses. (f) Distribution of hotspots of PD along each river, considering the six rivers individually.


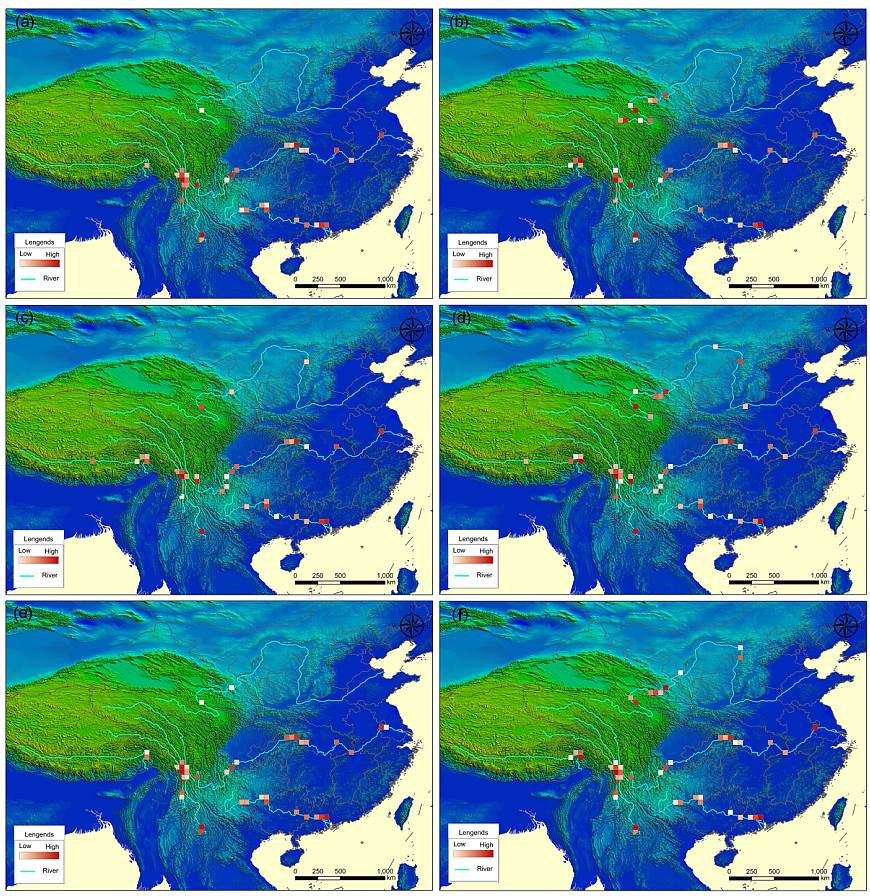


**Figure S7.** Violin plots showing the middle value of species richness, and the variation therein, in each of the six river valleys chosen in this study.

**
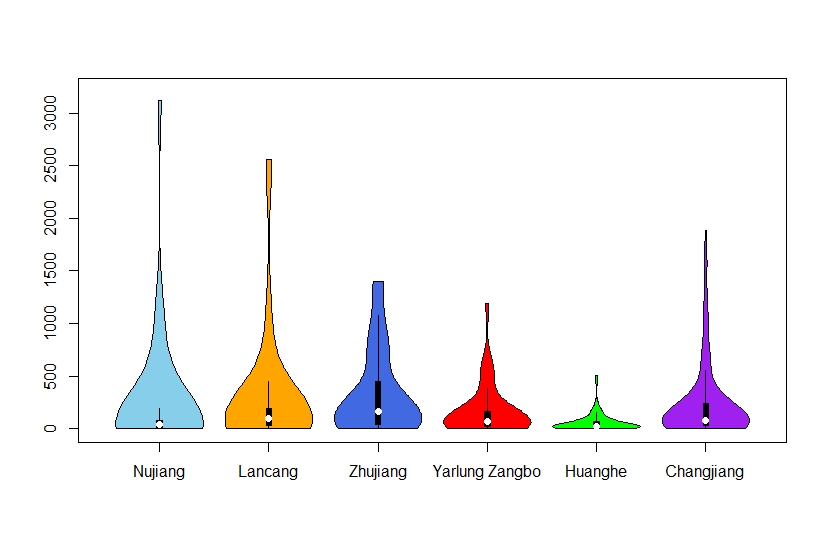
**

**Figure S8.** Chord diagram and circular barplot showing the number and area of grid cells and species composition of hotspots and conservation networks. The inner arcs link to the circular barplot by the same color to represent the group identity. The colored segments in the inner arc represent the number of species of specific groups, showing the variation in species numbers of the groups in different areas. The species composition for each groups (all species, threatened species, etc.) in the six LRVs is shown in the inner segments and the proportions of different groups are shown in the circular histogram on the outside.

**
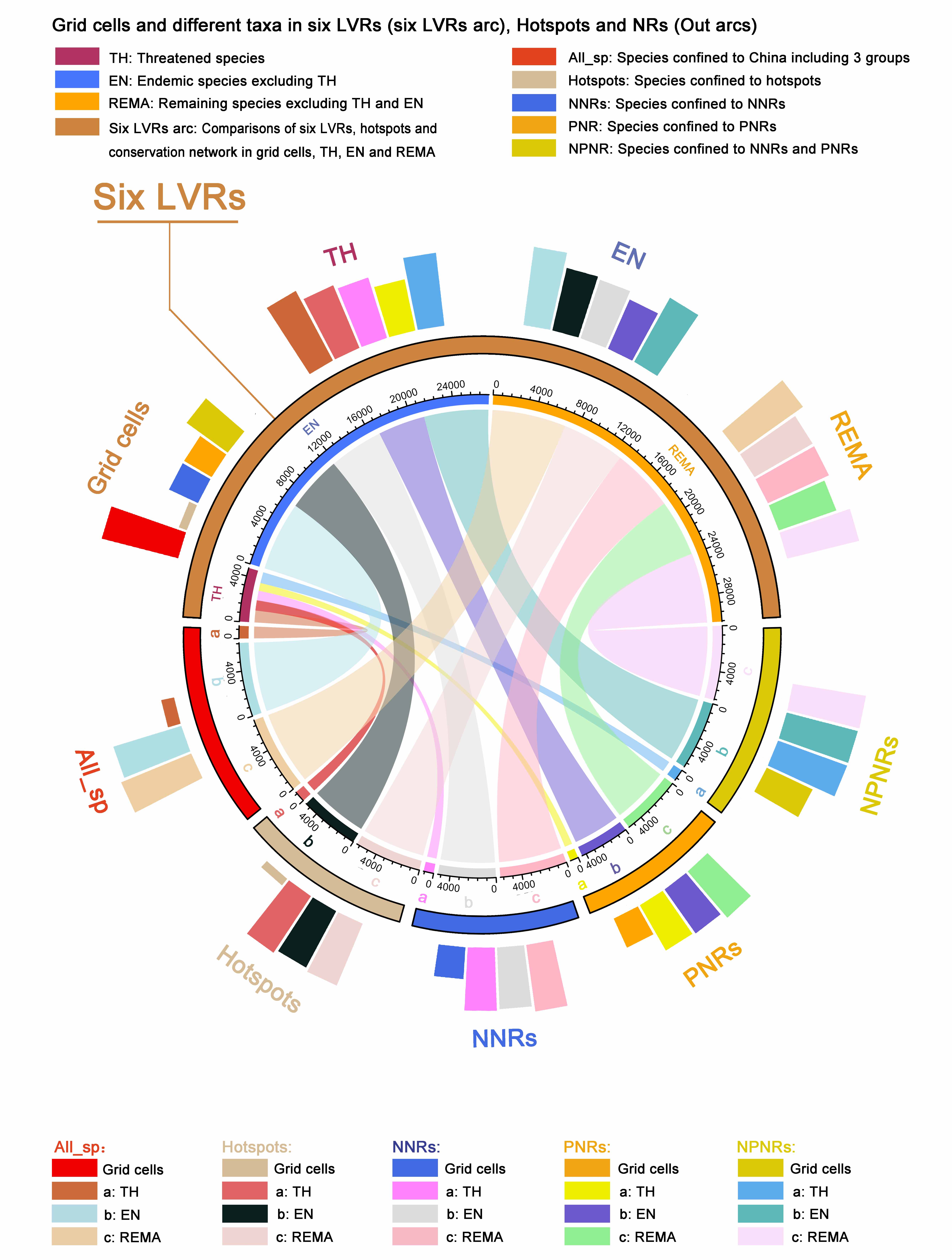
**

**Figure S9.** Chord diagram and circular barplot showing the number and area of hotspot grid cells and species composition of hotspots, conservation effectiveness and conservation gaps of NNRs and PNRs. The inner arcs link to the circular barplot by the same color to represent the same group. The colored fragments in the inner arc represent the number of species of different groups, or the aggregation of the hotspots, conservation effectiveness and conservation gaps for a given group. The ratio of the number of species of each group to the number of species of all species in hotspots is shown in All_sp.

**
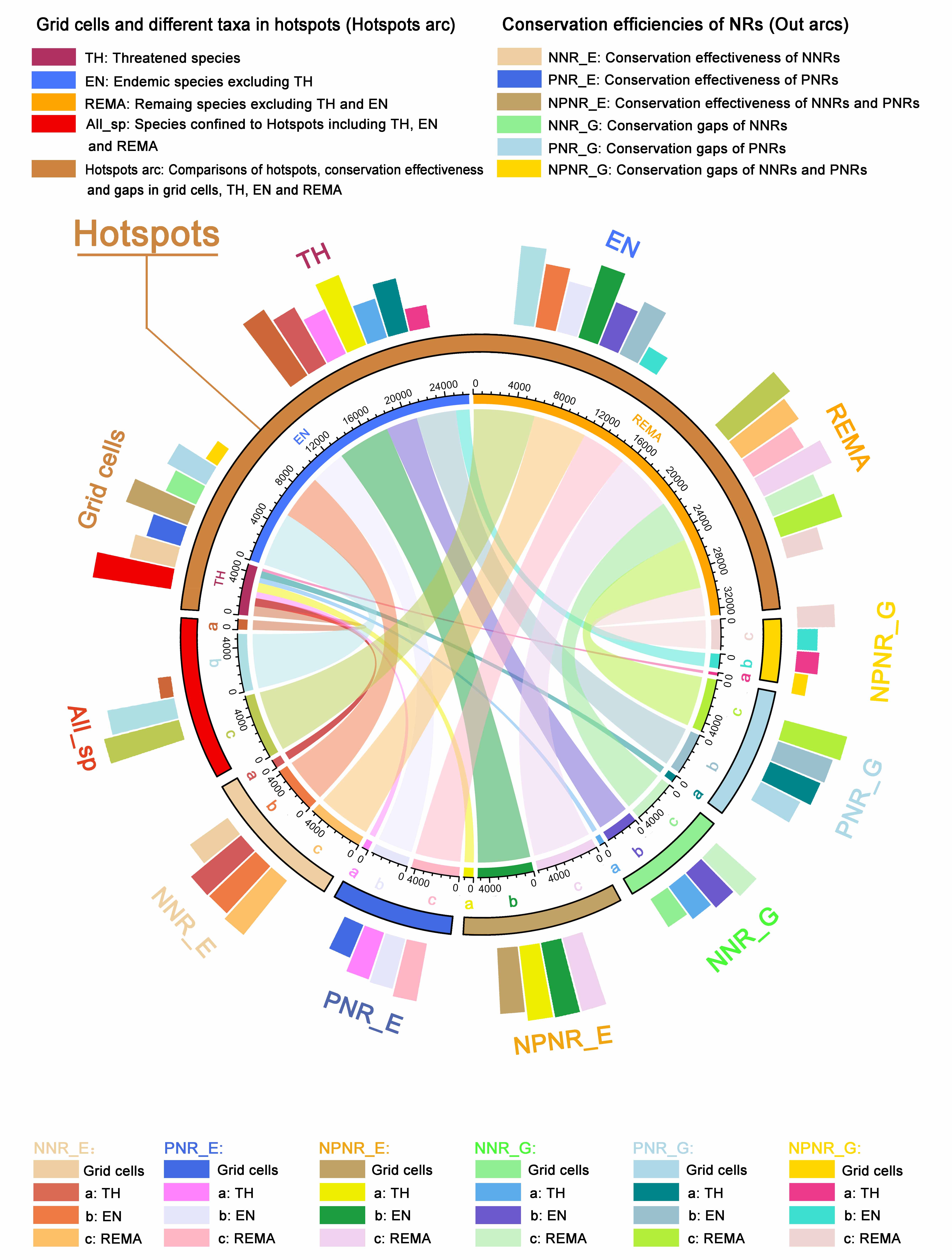
**
