# Supplementary material for: Insights into plant biodiversity conservation in large river valleys in China: A spatial analysis of species and phylogenetic diversity
Source: Ecol Evol. 2022 May 19;12(5):e8940. doi: 10.1002/ece3.8940 (PMC9120211; doi:10.1002/ece3.8940)
Supplement: Supplementary file 2 — Table S1 [file ECE3-12-e8940-s005.doc]

**Supporting information – Table S1**

**Table S1.1.** Key Features of six large rivers in China

| River | Length (km) | Drop (m) | Basin of area  (10000 km^2^) | Volume of runoff  (100000000 m^3^) |
| --- | --- | --- | --- | --- |
| Changjiang River | 6300 | 5400 | 180 | 9162 |
| Huanghe River | 5464 | 4830 | 79.5 | 534 |
| Lancang River | 4880 | 5167 | 81 | 4750 |
| Zhujiang River | 2214 | 2130 | 45.4 | 3319 |
| Nujiang River | 3673 | 4840 | 32.5 | 2525 |
| Yarlung zangbo River | 2057 | 5435 | 24.2 | 1661 |

**Table S1.2.** Species number observed in the six large valleys, conservation effectiveness and gaps in this study.

|  | Species number | | Species number of conservation network | | | Species number of conservation effectiveness | | | Species distributed in conservation gaps | | | Conservation gap species of hotspot | | |
| --- | --- | --- | --- | --- | --- | --- | --- | --- | --- | --- | --- | --- | --- | --- |
| Taxa | Six valleys | Hotspots | NNRs | PNRs | NNRs & PNRs | NNRs effectiveness | PNRs effectiveness | Effectiveness of NNRs & PNRs | NNRs gaps | PNRs gaps | Gaps of NNRs & PNRs | NNRs gaps | PNRs gaps | Gaps of NNRs & PNRs |
| All species | 14481 | 12081 | 11854 | 10468 | 13500 | 10206 | 8373 | 11608 | 7517 | 9295 | 4333 | 2063 | 3877 | 565 |
| Endemism | 7113 | 5778 | 5554 | 4799 | 6583 | 4681 | 3719 | 5553 | 3302 | 4166 | 1436 | 1091 | 2059 | 225 |
| Threatened | 1111 | 951 | 894 | 730 | 1034 | 783 | 594 | 904 | 483 | 657 | 271 | 168 | 357 | 47 |
| Nationally protected | 71 | 64 | 58 | 57 | 64 | 48 | 47 | 57 | 44 | 45 | 29 | 16 | 17 | 7 |

**Table S1.3.** Abundance of nationally protected, threatened (TH), endemic, endemic with threatened species excluded (EN), and the remaining species with TH and EN excluded (REMA), as a percentage of the total number of species in the study area and hotspots.

| Taxonomic groups | Species in six LRVs | Species in Hotspots | Variation |
| --- | --- | --- | --- |
| Nationally protected species | 0.49% | 0.53% | Up |
| Threatened species (TH) | 7.67% | 7.87% | Up |
| Endemic species | 49.12% | 47.83% | Down |
| Endemic species excluding threatened species (EN) | 44.71% | 43.37% | Down |
| Remaining species excluding TH and EN (REMA) | 47.62% | 48.65% | Up |
